# EML4–NTRK3 Fusion Cervical Sarcoma: A Case Report and Literature Review

**DOI:** 10.3389/fmed.2022.832376

**Published:** 2022-04-28

**Authors:** Xiaohe Dang, Tao Xiang, Can Zhao, Hao Tang, Pengfei Cui

**Affiliations:** ^1^Department of Obstetrics and Gynecology, Tongji Hospital, Tongji Medical College, Huazhong University of Science and Technology, Wuhan, China; ^2^Department of Pathology, Tongji Hospital, Tongji Medical College, Huazhong University of Science and Technology, Wuhan, China; ^3^Department of Radiology, Tongji Hospital, Tongji Medical College, Huazhong University of Science and Technology, Wuhan, China

**Keywords:** cervical sarcoma, NTRK gene fusion, case report, literature review, survival analysis

## Abstract

The concept of neurotrophic factor tyrosine kinase receptor (NTRK) fusion tumor has emerged in recent years. Moreover, NTRK fusion is unusual in common tumors but can often be identified in rare tumors. The NTRK fusion cervical or uterine tumors are mainly recognized through case reports due to their extremely low incidence. In this study, we reported a new case of EML4–NTRK3 fusion cervical sarcoma to enhance its recognition. To the best of our knowledge, this is the first case from a Chinese institution. We also conducted a literature review, in which a total of 19 cases of NTRK fusion cervical tumors and 4 cases of uterine tumors were retrieved. We summarized the clinicopathological features, treatment methods, and prognosis of these cases. Based on available information, we observed that surgery and complete excision, if possible, are still the primary modes of therapy. In addition, an increasing number of studies have shown that tropomyosin receptor kinases (TRK) inhibitors can improve the prognosis of cancer patients with NTRK gene fusion, which gives a silver lining for patients with metastatic lesions. We found that age and mitotic rate may be associated with recurrence or metastasis by univariate survival analysis. To draw more convincing conclusions, there is a need to establish an international database of rare cases and aggregate these sporadic cases.

## Introduction

The neurotrophic factor tyrosine kinase receptor (NTRK) gene family includes NTRK1, 2, and 3, which encode tropomyosin receptor kinases (TRK) A, B, and C, respectively (1). Accumulating evidence has revealed that NTRK fusion leads to a continuous elevation or activation of these TRK proteins. Meanwhile, these TRK family members have been demonstrated to induce cancer cell growth and activate downstream signaling pathways, such as PI3K-Akt-mTOR, Ras-MAPK-ERK, and PLC-γ-Ca2 + -PKC, that potentially lead to tumorigenesis (2–6). These findings have highlighted the role of NTRK fusion in the malignancy progression, and NTRK fusion genes have been actively investigated as promising therapeutic targets (7). Thus, it is essential to identify NTRK fusion in tumors.

Additionally, a recent study has revealed that NTRK fusion sarcoma is unique in pathological characteristics, treatment scheme, and prognosis compared with those without NTRK fusion (8). Therefore, to improve the recognition and treatment of this rare disease, in this study, we reported a new case of EML4–NTRK3 fusion cervical sarcoma, conducted a literature review to collect the existing reported cases of NTRK fusion uterine or cervical tumors, and summarized the clinicopathological features, treatment methods, and prognosis of these cases.

### Case Presentation

A 33-year-old Han Chinese woman was admitted to a local hospital due to irregular vaginal bleeding. The patient had no sexual history, so a transrectal ultrasound was performed instead of a gynecological examination, but no apparent abnormalities were found. So, the patient received hemostasis treatment but still experienced abnormal vaginal bleeding. Then, reexamination ultrasonography revealed a mass with rich blood supplies in the vagina, which had no clear boundary with the cervix, and subsequent magnetic resonance imaging (MRI) also found a 4.5 cm lesion filling the vagina, which was considered endometriosis or tumor. Then, the patient underwent hysteroscopy, and an elliptic mass was found at the cervical-vaginal junction. The mass was partially lobulated, with a smooth surface and a wide pedicle, and no noticeable abnormal vessels were observed. Later, the transvaginal cervical tumor resection was performed for the patient. Intraoperative investigation revealed that the tumor was in the anterior cervix lip and extended to the vaginal dome. It was pink, brittle, and had a rich blood supply. The histopathology of the excised specimen showed that the tumor tissue was composed of diffused spindle cells, and mitosis was active [>30/10 high power fields (HPFs)]. Collagen fiber degeneration around the vessels and lymphocyte infiltration were also observed, but necrosis was not detected. Immunohistochemical (IHC) staining was performed at the local hospital to differentiate it from other malignancies, such as high-grade endometrial stromal sarcoma or uterine leiomyosarcoma, and to determine its molecular subtype. It was found that the tumor was positive for S-100, CD34, vimentin, and cyclinD1 and negative for EMA, CK7, P53, P16, desmin, caldesmon, myoD1, myogenin, ALK, ER, PR, CD10, catenin-B, CD99, SATB2, BCL-2, CD117, BCOR, SOX10, HMB45, and melan-A. The tumor was suspected to be a special type of sarcoma, and then, the pathological sections were consulted by our hospital. We found that Pan-TRK, S-100, and CD34 were positive by IHC analysis, while SOX10 and desmin were negative in the tumor. Combined with its morphological characteristics and immunophenotype, it was suspected an NTRK fusion sarcoma. Since NTRK1 is the most common fusion type, it was investigated by fluorescence *in situ* hybridization (FISH) assay. The results showed that approximately 11% of cells were 0r-1Gr-1Fu, which did not support the existence of NTRK1 rearrangement (NTRK1 gene signal was mostly 1-3FU) in the tumor (Supplementary Figure 1).

**FIGURE 1:**
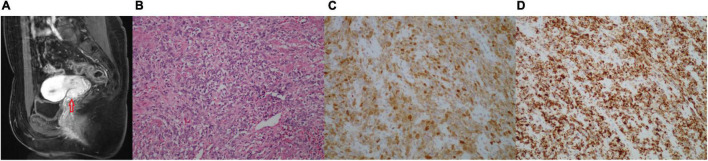
**(A–D)** Images of the cervical tumor. **(A)** The pelvic MRI (sagittal T1-weighted images with contrast) showing a 1.8 × 1.5 × 1.3 cm abnormal signal in the anterior wall of the cervical-vaginal junction, which was considered a neoplastic lesion. **(B)** H&E-stained (200×) of the cervical tumor: the tumor tissue was composed of diffused spindle cells, arranged in crisscross bundles, sheets, or zigzag, and many blood vessels were seen and had low to moderate grade nuclear atypia. **(C)** Immunohistochemical (IHC) of S-100 (200×) was positive. **(D)** IHC of Pan-TRK (200×) was strongly positive.

The patient was transferred to our hospital for further treatment. The MRI in our hospital showed a neoplastic lesion on the anterior wall of the cervical–vaginal junction (Figure 1A). The serum levels of tumor markers were as follows: HE4 25.2 pmol/L, CA125 62.9 U/ml↑, SCC 0.8 ng/ml, β-HCG <0.1 mIU/ml, AFP 1.92 ng/ml, CEA 0.71 ng/ml, CA199 7.27 U/ml, and NSE 12.17 μg/L. Meanwhile, a hysteroscopy examination was performed to distinguish it from endometrial stromal sarcoma. During the operation, a 0.5 × 1.5 cm neoplasm was found on the posterior wall of the uterus, and the pathological examination revealed an endometrial polyp (Supplementary Figure 2). Then, the patient underwent an extensive laparoscopic hysterectomy, bilateral salpingo-oophorectomy, pelvic lymph node dissection, and partial vaginal resection. The histopathology revealed the macroscopic feature of the tumor: the tumor tissue was composed of diffused spindle cells with fascicular, nested, or uniform patterns, and vascularization and nuclear atypia (low to moderate) were observable (Figure 1B). Meanwhile, there was no lymph node metastasis, lymph vascular space invasion (LVSI), and parauterine/interstitial infiltration. Furthermore, IHC analysis revealed that the Ki-67 labeling index (LI) was 30–40%, indicating highly proliferative tumor cells, and a large portion of tumor cells strongly expressed Pan-TRK, S-100, and CD34, while negative for vimentin, SOX10, and desmin (Figures 1C,D). Consequently, based on the morphological and IHC results, we diagnosed the patient as NTRK fusion cervical sarcoma. According to the 2018 Federation International of Gynecology and Obstetrics (FIGO) staging system for cervical cancer, the stage of the patient was IIA2. Postoperatively, the patient received 6 courses of adjuvant chemotherapy with doxorubicin hydrochloride liposomes and nedaplatin. On the completion of chemotherapy, the patient received pelvic MRI and abdominal computed tomography (CT) examinations, and the results showed no abnormalities. Meanwhile, she visited our hospital to monitor the CA125 level in the next 3 months, which were 46.3 U/ml↑, 25.7 U/ml, and 20.5 U/ml, respectively.

**FIGURE 2:**
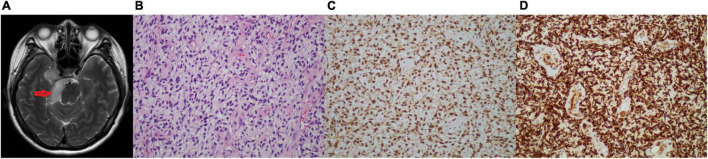
**(A–D)** Images of the brain tumor. **(A)** The head MRI (axial showed images) showing a 4.6 × 2.2 cm neoplastic lesion in the trigeminal area. **(B)** H&E-stained (200×): the tumor tissue is composed of homogenous spindle cells, arranged in bundles, swirls, and sheets, mitotic activity, and rich blood vessels. **(C)** IHC of S-100 (200×) was positive. **(D)** IHC of Pan-TRK (200×) was strongly positive.

**FIGURE 3:**
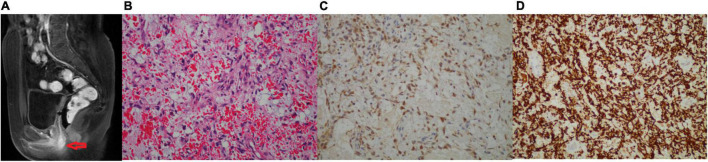
**(A–D)** Images of the vagina tumor. **(A)** The pelvic MRI (sagittal T1-weighted images with contrast) showing homogenous enhancement from the posterior vagina to the vulva with approximately 1.5 × 1.6 cm, which was considered tumor recurrence. **(B)** H&E-stained (200×): the tumor tissue is composed of predominantly monomorphic spindled cells arranged in bundles, sheets, or zigzag. The nuclei are oval, chromatin vacuolated, nucleoli are not evident, mitosis is easy to see, and no necrosis. **(C)** IHC of S-100 (200×) was positive. **(D)** IHC of Pan-TRK (200×) was strongly positive.

Two months after her last follow-up, the patient visited our hospital for diplopia in the right eye and right facial numbness, and the MRI examination revealed a mass in the right trigeminal nerve area, which was considered trigeminal schwannoma (Figure 2A). Consequently, she underwent cerebellopontine angle lesion resection in the Neurosurgery Department. During the operation, it was found that the tumor was, in fact, undemarcated with the trigeminal nerve. Grossly, the tumor was gray and fragmentary. Microscopic examination revealed that the tumor was spindle cell sarcoma (Figure 2B), which was diffusely expressed of CD34, vimentin, S-100, and Pan-TRK (Figures 2C,D), but negative for SOX10, EMA, SSTR2, PR, and STAT6. More importantly, the next-generation sequencing (NGS) identified an EML4 (NM_019063.5: exon 2)-NTRK3 (NM_001012338.2: exon 14) gene fusion variation in the tumor tissue. Together, the IHC and NGS results confirmed that the patient had a spindle cell sarcoma with EML4–NTRK3 fusion in the middle cranial fossa. To assess the condition of the lungs, the patient received the chest CT scan, which showed multiple metastatic lesions in both lungs, and the largest lesion was approximately 1.9 × 2.4 cm (Supplementary Figure 3). One month later, the patient presented vaginal bleeding, a gynecological examination found a mass protruding from the vaginal external orifice, and MRI revealed a 1.5 × 1.6 cm mass from the posterior of the vagina to the vulva, which was considered tumor recurrence (Figure 3A). The patient suffered from bleeding due to the mass and underwent lesion resection. Microscopically, the resected specimen was also composed of spindle cells (Figure 3B) and showed strongly positive for Pan-TRK, S-100, CD34, and vimentin but negative for SOX10 and desmin (Figures 3C,D). The results pointed to the recurrence and invasion of the NTRK rearrangement tumor. Also, the diagnosis indicated that the patient might benefit from TRK inhibitors, which, unfortunately, were not widely available in China currently. We sought a clinical trial of TRK inhibitor for this patient, but her condition progressively worsened and presented drowsiness, salivation, and left leg weakness. Unfortunately, the patient passed away one month later without being enrolled in the clinical trial.

### Literature Review and Survival Analysis

We searched the China National Knowledge Infrastructure (CNKI), PubMed, and MEDLINE databases to retrieve the reported cases of NTRK gene fusion cervical or uterine tumors. In addition, the retrieval time was from the establishment of the database to June 2021, and the search term was “NTRK.” Finally, a total of 11 articles with 23 cases were included. The literature search flowchart is shown in Supplementary Figure 4. There were only 19 cases of NTRK fusion cervical tumors and 4 cases of uterine tumors documented, and the most common gene fusion type was TPM3-NTRK1 (11/23). The clinical presentations included irregular vaginal bleeding, prolonged menstruation, cervix neoplasm, and uterine fibroids. However, some cervical tumors had been detected during the screening test while the patients were symptomless. Histologically, most reported tumors were sarcoma, and only case 23 with cervical stump carcinoma was basaloid squamous cell carcinoma. In addition, most tumors showed mild to moderate grade nuclear atypia, and a few tumors exhibited necrosis and active mitosis. Moreover, Pan-TRK was positive in all tumors, and S100 was positive in all the tumors except cases 5 and 23. Six patients developed metastasis or recurrence, and the metastatic sites included vagina, lung, pleura, brain, pancreas, omentum, and ovary. Additionally, imaging examinations in cases 2 and 23 indicated pulmonary metastasis at their first visit. Moreover, the shortest duration for tumor recurrence was only 7 months (Case 2). Additionally, the maximum follow-up time was 108 months (Case 12), when the patient was still surviving with no sign of tumor relapses. Two patients died: one patient died 78 months after onset, the other patient (our case) died approximately 14 months after the tumor was detected. Information about the first author, country, publication time, clinical manifestations, imaging features, tumor size, patient age, tumor site, and gene fusion type of the included cases are summarized in Supplementary Appendix Table 1. In addition, the disease stage, treatment methods, macroscopic and microscopic features of tumor tissue, immunophenotypic features, and prognosis of the 24 patients are described in Supplementary Appendix Table 2 (8–19).

**FIGURE 4:**
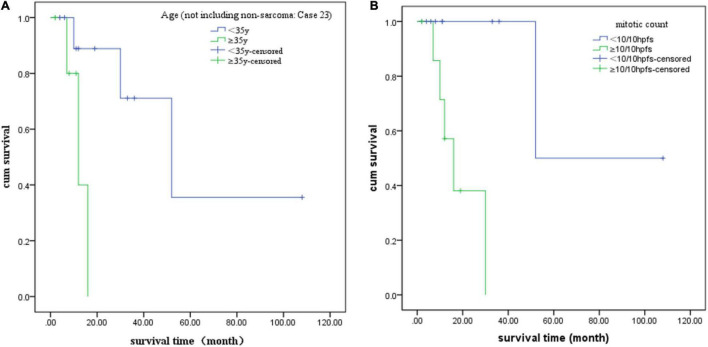
Kaplan–Meier analysis curves of each statistically significant factor. **(A)** Age and **(B)** mitotic count.

We also analyzed potential prognostic factors based on the 18 patients with prognostic data. The starting point was the first diagnosis, and the endpoint was tumor recurrence or metastasis. The survival time was presented in months. SPSS software (version 22.0) was used to perform statistical analysis, and *P* < 0.05 was considered statistically significant. Kaplan–Meier and log-rank tests were used for univariate survival analysis (20). As case 23 was a special cancer, she was not included in the univariate survival analysis to reduce bias. We found that age ≥ 35 years (*P* = 0.016) and mitosis count ≥ 10/10 HPFs (*P* = 0.009) were related to tumor recurrence or metastasis. However, tumor ≥ 5 cm in diameter (*P* = 0.982), NTRK fusion type (*P* = 0.147), necrosis (*P* = 0.264), atypia (*P* = 0.156), and oophorectomy (*P* = 0.067) were not statistically significant, which may be attributed to the cohort size. The log-rank survival analysis results of each factor are summarized in Table 1, and the Kaplan–Meier analysis results of significant factors are presented in Figure 4.

**TABLE 1:** Univariate survival analysis.

Factor	Case (patient number)	Recurrent group-n	Non-recurrent group-n	X^2^	*P*-value
Age				5.772	0.016
<35 y	5, 6,9,10,11,12,13,16,17,18,24	3	8		
≥35 y	2,3,4,8,19,20,22	3	4		
Size (not including uterine tumors)				0.001	0.982
<5 cm	4, 6,8,9,10,11,17,18,19,22,24	3	8		
≥5 cm	2,3,12,13,20	3	2		
Gene fusion type				2.098	0.147
TPM3-NTRK1	4, 6,8,9,10,11,12,17,20	1	8		
Non-TPM3-NTRK1	2,3,5,13,16,18,19,22,24	4	5		
Necrosis				1.246	0.264
Yes	2,3,9,11,13	4	1		
No	4,5,6,8,10,12,16,17,18,19,20,22,24	2	11		
Atypia				2.016	0.156
Mild	10,11,12,16	1	3		
Moderate or severe	2,3,4,5,6, 8,9,13,17,18,19,22,24	5	8		
Mitotic count				6.884	0.009
<10/10 hpfs	5,6,8,9,12,13,16,17, 20,22	1	9		
≥10/10 hpfs	2,3,4,10,11,18,19,24	5	3		
Oophorectomy				3.364	0.067
No	6,17,18,19,20	1	4		
Yes	2,3,4,5,22,24	3	3		

## Discussion

In this study, we reported a case of EML4–NTRK3 fusion cervical sarcoma. To the best of our knowledge, this patient was the first reported case from a Chinese institution. In addition, we also conducted a literature review to summarize the clinicopathological features, treatment methods, and prognosis of the NTRK fusion cervical or uterine tumor and found that age and mitotic rate were associated with metastasis by univariate survival analysis. Moreover, the diagnostic methods, therapeutic measures, and prognostic factors of this disease were discussed in the following paragraphs.

The diagnosis of NTRK fusion cervical or uterine sarcoma is difficult, requiring advanced molecular pathology techniques (21) and a long time. The predominant symptoms are not specific and similar to other tumors (22), including irregular vaginal bleeding, cervical mass, or uterine fibroids. The pathological characteristics of NTRK fusion sarcoma are more apparent (23): the tumor is usually composed of diffused spindle cells (24). However, the morphological features cannot distinguish it from other rare tumors such as leiomyosarcoma and high-grade endometrial stromal sarcoma (25). Thus, IHC analysis is required for differential diagnosis. Moreover, it has been found that NTRK fusion tumors are generally positive for S100 and Pan-TRK and negative for SOX10, desmin, vascular markers, and various keratin proteins (26). However, the smooth muscle markers are positive in leiomyosarcoma (27), and BCOR is positive in high-grade endometrial stromal sarcoma (28). The IHC results of this patient supported the diagnosis of NTRK fusion sarcoma. Therefore, we proposed that if the sarcoma is composed of diffused spindle cells and the expression of various tissue factors is negative, the possibility of NTRK gene fusion should be considered, and Pan-TRK staining should be added (29). NTRK fusion triggers the activation and overexpression of TRK, which has high sensitivity and specificity for the diagnosis of NTRK fusion sarcoma. However, other tumors without NTRK fusion can also display positive Pan-TRK expression. For example, Chiang et al. (10) found that uterine leiomyosarcoma without NTRK gene fusion also expressed Pan-TRK. Meanwhile, Cordier et al. (30) found that rhabdomyosarcoma with YAP1-MAML2 fusion showed Pan-TRK overexpression. Consequently, the detection of NTRK fusion still requires molecular pathology analysis, such as NGS, RT-PCR, and FISH (31).

Most of the reported patients underwent surgery and complete excision, and some patients received adjuvant radiotherapy, chemotherapy, and TRK inhibitors postoperatively. However, Chin et al. (32) found that partial cervical resection was feasible for pedicled sarcoma in the cervix, which could preserve fertility for young women. Among the 13 reported cases with known surgical procedures, only 2 patients underwent partial cervical resection: one was a young patient with a cervix polypoid mass, while the other was an elderly female with cervical stump carcinoma. More studies are required to determine the feasibility and safety of uterine retention. In addition, we noticed that 4 out of 5 patients with their ovaries retained had no metastasis during the follow-up, and the longest follow-up duration was 19 months. Meanwhile, pleural metastasis was observed in one patient during follow-ups at 16 months. The patient we reported was at stage IIA2, and the tumor cells were poorly differentiated, actively mitotic, and with a high degree of malignancy. Moreover, the biological invasiveness of the NTRK fusion sarcoma is still not fully clear. We thought that the patient was at a high risk of metastasis and resected her ovaries. In addition, some studies showed that not all cervical cancers need ovariectomy. For instance, Chen et al. (33) suggested that ovarian preservation could be considered for cervical adenocarcinoma if the patients are not exposed to ovarian metastasis risk factors. Also, Matsuo et al. (34) found that in patients with stage I cervical cancer (not including sarcoma or metastasis tumor), the risk of metachronous ovarian cancer after ovarian preservation was <1%. Considering that most patients are relatively young, it remains to be investigated whether NTRK fusion cervical or uterine sarcoma requires ovariectomy. In addition, the patient needed adjuvant radiotherapy or chemotherapy postoperatively. According to the American Society for Radiation Oncology evidence-based guideline (35), radiotherapy is not the first choice for sarcomas. Therefore, we finally chose chemotherapy for the patient. Despite that imaging examinations did not indicate disease progression during chemotherapy, multiple metastases were identified sooner after treatment, indicating that the patient had a poor response to chemotherapy. Therefore, further research is needed to determine an appropriate postoperative treatment for this malignancy. Fortunately, studies have shown that TRK inhibitors such as entrectinib and larotrectinib (36, 37) are safe and effective in NTRK fusion tumors (7). Clinical trials have demonstrated that larotrectinib and entrectinib are suitable for a long-term administration with few side effects (36, 38, 39). In our review, cases 19 and 23 were provided with larotrectinib for postoperative treatment, which suppressed the metastases and without noticeable side effects. However, TRK inhibitors are not currently common in China, and the patient passed away without being enrolled in a clinical trial of TRK inhibitor. In addition, TRK inhibitors are scarce in developing regions, and these patients have no access to get these targeted drugs (40). We think that international collaborative drug clinical trials are necessary, which may provide an opportunity for targeted therapy for patients with NTRK fusion tumors who continue to deteriorate after conventional therapy.

We found that patients with age ≥ 35 years and mitosis count ≥ 10/10 HPFs may be associated with tumor recurrence or metastasis based on univariate survival analysis. Therefore, we suggested that the follow-up should be more frequent for such patients, and the patient’s condition, detailed physical examination, and necessary imaging examination should be thoroughly evaluated to detect tumor metastasis and recurrence. The case we reported presented neurological symptoms at the time of recurrence, and MRI found a mass in the right trigeminal nerve area. We think that the medical history is critical, and the possibility of the metastatic tumor should be considered when patients with tumor history develop tumors at other sites. Another patient (case 3), a 47-year-old woman with LMNA-NTRK1 fusion, also developed central nervous system (CNS) metastasis and died 78 months after the onset. CNS metastasis is an important cause of poor prognosis. Hong et al. (36) found that most tumors with NTRK fusion were not prone to CNS metastasis, except lung cancer, thyroid cancer, and melanoma. However, case 3 and the current case demonstrate that NTRK fusion cervical sarcoma could also develop CNS metastasis. Of note, NTRK1 overexpression has been linked with CNS metastasis in breast cancer (41). In this study, we reported that NTRK3 overexpression may also be associated with CNS metastasis in cervical or uterine sarcomas. In addition, NTRK3 or NTRK1 fusion genes are associated with distant metastasis in thyroid carcinomas (6). Our study found no significant difference in metastases between the most common TPM3-NTRK1 fusion and other fusion types in cervical or uterine sarcomas. Moreover, cases 2, 3, and 23 and our patient all had pulmonary metastasis, so the lung may be a common distant metastatic site for this type of tumor. In addition, we observed that different staging methods had been used in different cases. It remains to be studied what the staging method should be used for cervical sarcoma in the future to standardize the staging method of the disease. Although CA125 has been widely used as a prognostic marker for ovarian epithelial carcinoma (42) and endometrial carcinoma (43), there is insufficient evidence that CA125 is a prognostic marker for sarcoma (44). Our patient’s CA125 level was higher than normal preoperatively, and the postoperative follow-up showed that her CA125 level decreased, but the disease progressed. The increase of CA125 may be related to endometriosis, inflammation, and other physiological and pathological factors (43). These observations further indicate that CA125 is not a prognostic marker for NTRK fusion cervical sarcoma. Tumor markers for patients with sarcoma should be further explored, and exosomes may be used as tumor markers for sarcoma in the future (45). Due to the rarity of the cases, the prognostic factors of NTRK fusion cervix or uterus sarcoma are still ambiguous, and more data are needed to draw a solid conclusion.

### Limitations

There are some limitations in our study: (1) due to the dispersion of cases, the complete patients’ data are difficult to obtain accurately. (2) Our univariate survival analysis may not fully reflect the characteristics of the disease due to the small number of cases, and more data are needed for further confirmation. (3) There are no unified guidelines for diagnosis and treatment as a reference for writing this manuscript. Therefore, regional and international cooperation is needed to conduct more research and establish guidelines for diagnosing and treating these rare diseases.

## Conclusion

We reported a case of EML4–NTRK3 fusion cervical sarcoma. To the best of our knowledge, this is the first case that came from a Chinese institution. Moreover, traditional surgery and chemotherapy had no significant effect on this patient, her condition deteriorated rapidly, and she passed away only 14 months after onset. Based on available information, we noticed that surgery and complete excision, if possible, are still the main modes of therapy. In addition, an increasing number of studies have shown that TRK inhibitors can improve the prognosis of cancer patients with NTRK gene fusion, which gives a silver lining for patients with metastatic lesions. Through univariate survival analysis, we found that age and mitotic rate may be associated with recurrence or metastasis. To draw more convincing conclusions, it is necessary to establish an international database of rare cases and aggregate these sporadic cases.

## Data Availability Statement

The original contributions presented in the study are included in the article/Supplementary Material, further inquiries can be directed to the corresponding author.

## Ethics Statement

The manuscript is a case report with a literature review. No human or animal studies were carried out in preparing the manuscript. Ethical review and approval were not required for the study in accordance with the local legislation and institutional requirements.

## Author Contributions

XD, TX, and PC conceived the idea, wrote, and revised the manuscript. CZ analyzed the histopathological figures. HT analyzed the radiological figures. All authors approved the final manuscript.

## Conflict of Interest

The authors declare that the research was conducted in the absence of any commercial or financial relationships that could be construed as a potential conflict of interest.

## Publisher’s Note

All claims expressed in this article are solely those of the authors and do not necessarily represent those of their affiliated organizations, or those of the publisher, the editors and the reviewers. Any product that may be evaluated in this article, or claim that may be made by its manufacturer, is not guaranteed or endorsed by the publisher.

## Supplementary Material

Supplementary Figure 1 Representative image of the fluorescence *in situ* hybridization (FISH) assay evaluating the expression of NTRK1 in the tumor tissue (A: 100×, B: 400×): the NTRK1 rearrangement was shown by a paired red (centromeric)–green (telomeric) fluorescence signal, NTRK1 gene signal was mostly 1-3FU, but only approximately 11% of cells were 0R-1GR-1FU in this case, which did not support the existence of NTRK1 rearrangement in the tumor.

Supplementary Figure 2 Images of H&E-stained (100× and 400×) of the endometrial polyp.

Supplementary Figure 3 Image of the chest computed tomography (CT) showing multiple nodules in both lungs, and the largest one was 1.9 × 2.4 cm (red arrowhead on the right).

Supplementary Figure 4 Flowchart for the literature.
