# Supplementary figures and images for: EML4–NTRK3 Fusion Cervical Sarcoma: A Case Report and Literature Review
Source: Front Med (Lausanne). 2022 Apr 28;9:832376. doi: 10.3389/fmed.2022.832376 (PMC9096266; doi:10.3389/fmed.2022.832376)

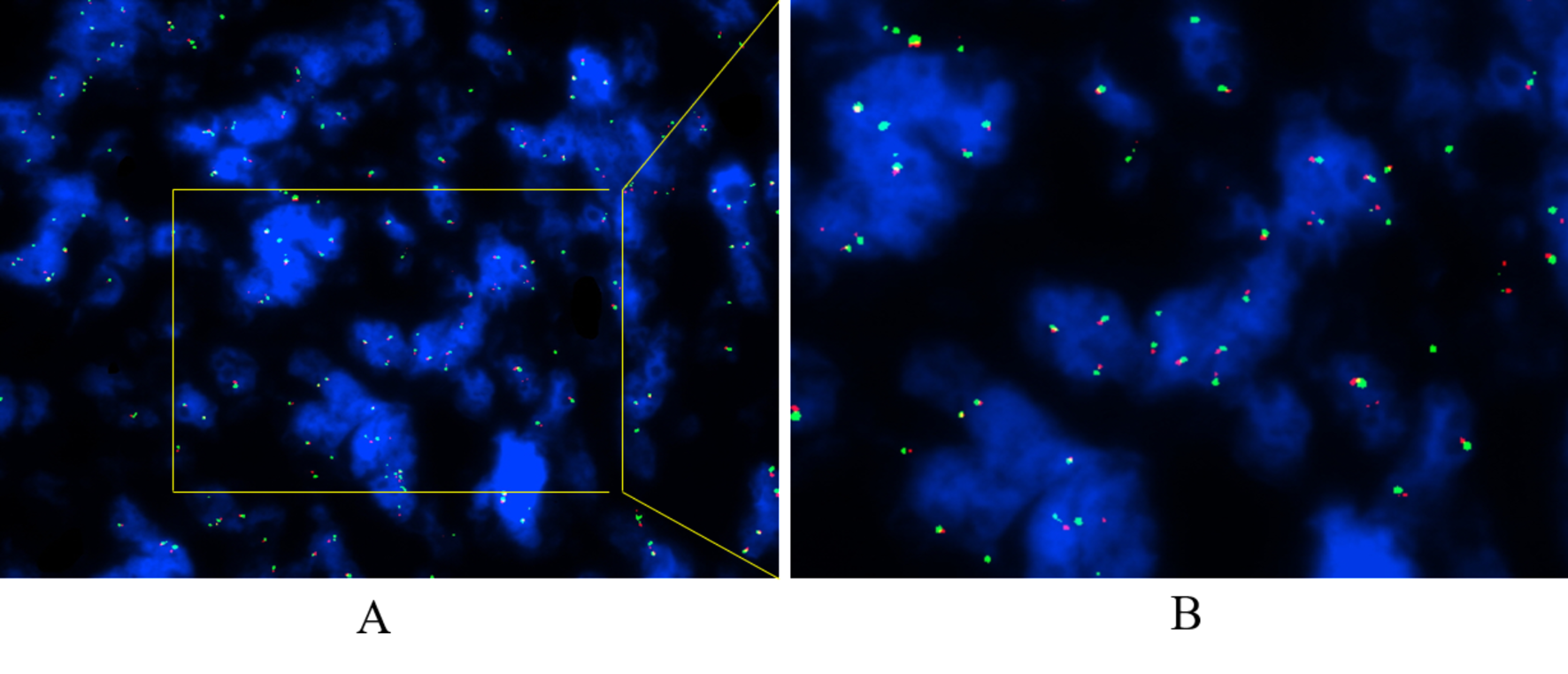

Supplement: Supplementary Figure 1 — Representative image of the fluorescence in situ hybridization (FISH) assay evaluating the expression of NTRK1 in the tumor tissue (A: 100×, B: 400×): the NTRK1 rearrangement was shown by a paired red (centromeric)–green (telomeric) fluorescence signal, NTRK1 gene signal was mostly 1-3FU, but only approximately 11% of cells were 0R-1GR-1FU in this case, which did not support the existence of NTRK1 rearrangement in the tumor. [file Image_1.TIFF]

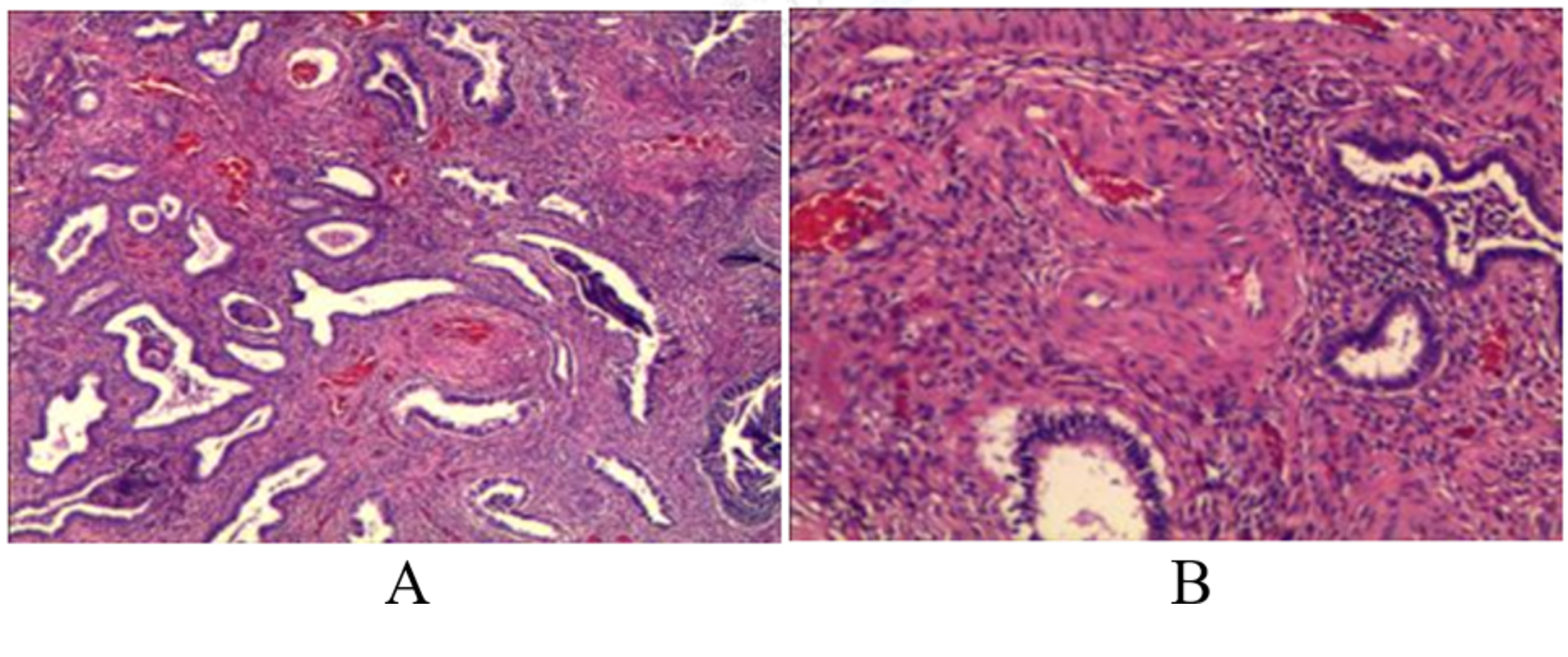

Supplement: Supplementary Figure 2 — Images of H&E-stained (100× and 400×) of the endometrial polyp. [file Image_2.TIFF]

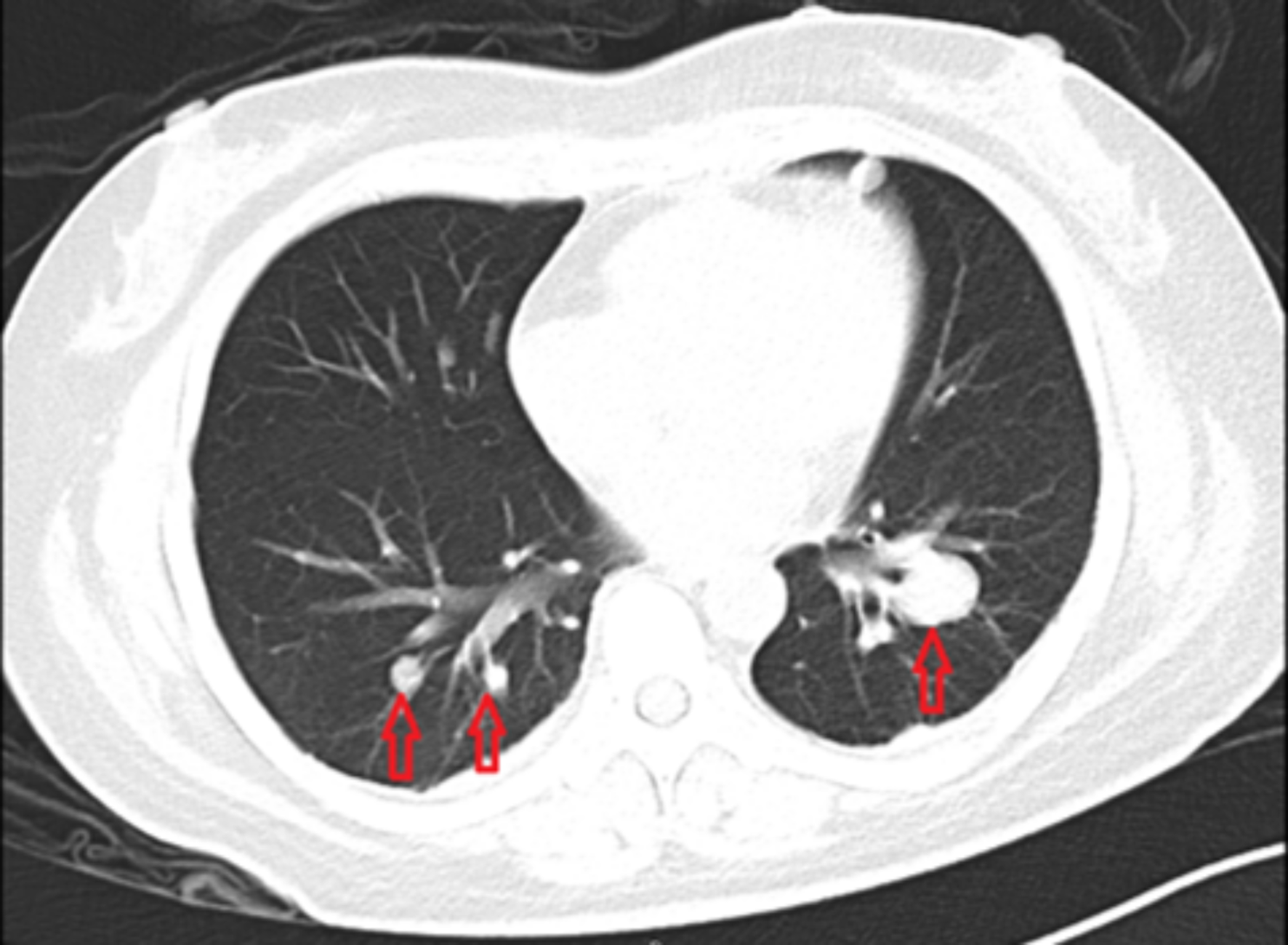

Supplement: Supplementary Figure 3 — Image of the chest computed tomography (CT) showing multiple nodules in both lungs, and the largest one was 1.9 × 2.4 cm (red arrowhead on the right). [file Image_3.TIFF]

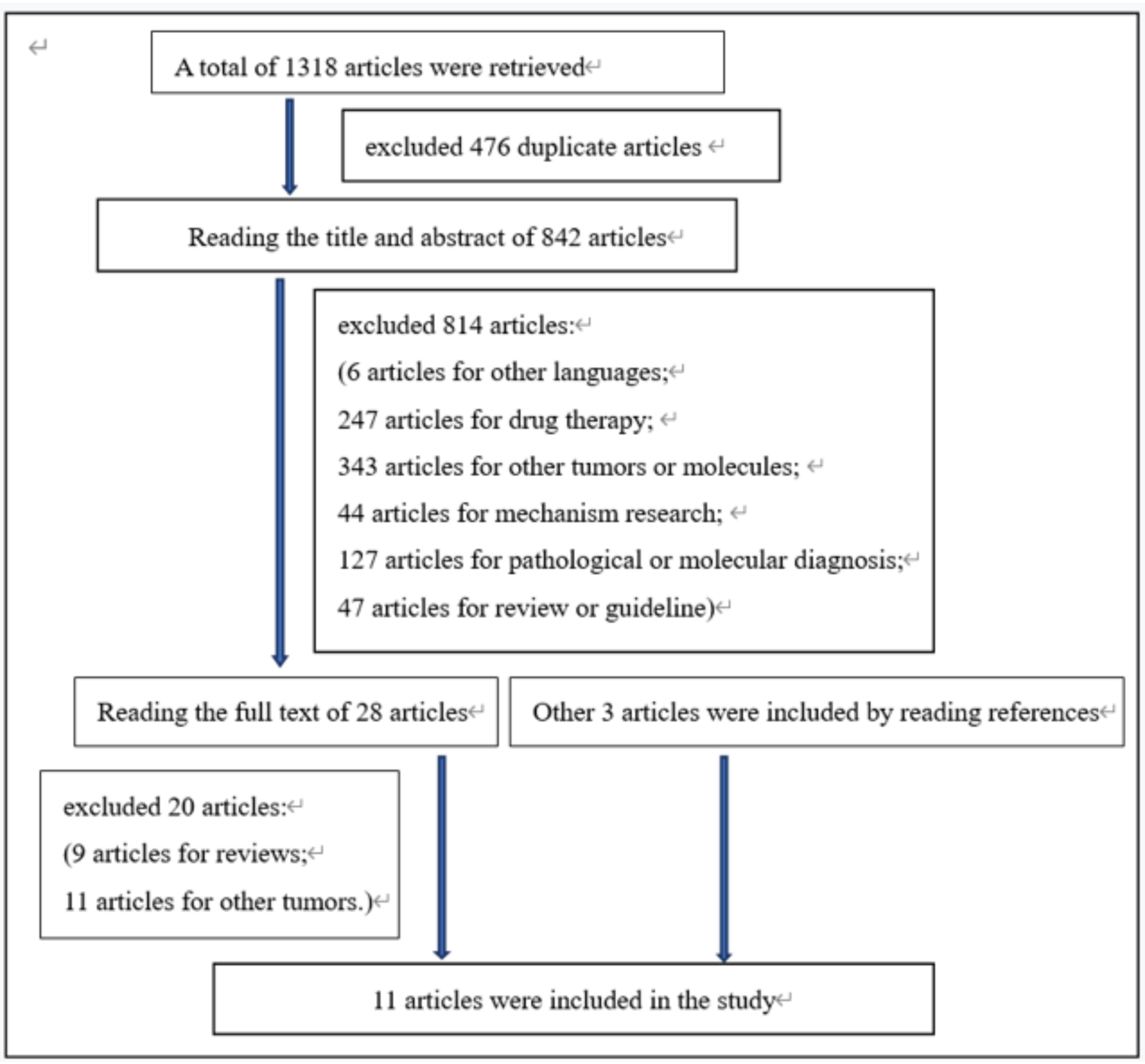

Supplement: Supplementary Figure 4 — Flowchart for the literature. [file Image_4.TIFF]
