# Supplementary material for: EML4–NTRK3 Fusion Cervical Sarcoma: A Case Report and Literature Review
Source: Front Med (Lausanne). 2022 Apr 28;9:832376. doi: 10.3389/fmed.2022.832376 (PMC9096266; doi:10.3389/fmed.2022.832376)
Supplement: Supplementary file 5 [file Data_Sheet_1.docx]

Appendix table 1. General characteristics of the included articles and cases.

| **First author (year)** | **Country** | **Case (number)** | **Age (year)** | **Clinical Presentations** | **Tumor site** | **Imaging presentations** | **Size (cm)** | **Gene fusion type** |
| --- | --- | --- | --- | --- | --- | --- | --- | --- |
| Hartmaier et al. (2017) | America | 1 | NA | NA | uterus | NA | NA | TPR-NTRK1 |
| Chiang et al. (2018) | America | 2 | 46 | Vaginal bleeding+ cervix neoplasm | cervix | Suspicious metastatic nodules in the lungs | 9.3 | RBPMS-NTRK3 |
|  |  | 3 | 47 | Vaginal bleeding+ cervix neoplasm | cervix | NA | 14 | LMNA-NTRK1 |
|  |  | 4 | 42 | Vaginal bleeding+ cervix neoplasm | cervix | NA | 2.6 | TPM3-NTRK1 |
|  |  | 5 | 27 | Vaginal bleeding + uterine fibroids | uterus | Uterine fibroids | 16.3 | TPR-NTRK1 |
| Wells et al. (2019) | America | 6 | 30 | Asymptomatic, detected during cervical cancer screening | cervix | Cervix neoplasm | 2.5 | TPM3-NTRK1 |
| Croce et al. (2019) | France | 7 | 39 | NA | cervix | NA | NA | TPM3-NTRK1 |
|  |  | 8 | 44 | NA | cervix | NA | 4.5 | TPM3-NTRK1 |
|  |  | 9 | 23 | NA | cervix | NA | 3 | TPM3-NTRK1 |
|  |  | 10 | 30 | NA | cervix | NA | 2.5 | TPM3-NTRK1 |
|  |  | 11 | 23 | NA | cervix | NA | 2.8 | TPM3-NTRK1 |
|  |  | 12 | 33 | NA | cervix | NA | 5 | TPM3-NTRK1 |
|  |  | 13 | 26 | NA | cervix | NA | 12 | EML4-NTRK3 |
| Gatalica et al. (2019) | America | 14 | NA | NA | cervix | NA | NA | TPM3-NTRK1 |
|  |  | 15 | NA | NA | uterus | NA | NA | SPECC1L-NTRK3 |
| Michal et al. (2019) | Australia | 16 | 26 | Uterine fibroids | uterus | NA | 23 | STRN-NTRK3 |
| Rabban et al. (2020) | America | 17 | 24 | Vaginal bleeding+ cervix neoplasm | cervix | Cervix neoplasm | 1.5 | TPM3-NTRK1 |
|  |  | 18 | 30 | Vaginal bleeding+ cervix neoplasm | cervix | Cervix neoplasm | 1.8 | TPR-NTRK1 |
|  |  | 19 | 49 | Asymptomatic, detected during cervical cancer screening | cervix | Cervix neoplasm | 4.7 | SPECC1L–NTRK3 |
| Boyle et al. (2020) | England | 20 | 42 | Vaginal bleeding+ cervix neoplasm | cervix | NA | 5.2 | TPM3-NTRK1 |
| Wong et al. (2020) | Australia | 21 | 31 | Cervix neoplasm | cervix | NA | 9 | NTRK3 |
| Hodgson et al. (2021) | Canada | 22 | 60 | Chronic menorrhagia | cervix | Myoma and adenomyosis | 1.6 | SPECC1L-NTRK3 |
| Munkhdelger et al. (2021) | Japan | 23 | 72 | Vaginal bleeding+ cervix neoplasm | cervix | Cervix neoplasm+ multiple metastatic lesions in both lungs | 6 | DLG2-NTRK2 |
| This case | China | 24 | 33 | Vaginal bleeding+ cervix neoplasm | cervix | Cervix neoplasm | 4.5 | EML4-NTRK3 |

NA：not available in the original article. The median age was 33 years old (age information was not available for 3 patients). The mean diameter of uterine tumors and cervical tumors was 19.7 cm and 5.1 cm, respectively (diameter information was not available in 4 patients).

Appendix table 2. Pathological features, treatment methods and prognosis of the included cases.

| **Case**  **(number)** | **Stage** | **Treatment** | **Macroscopic** | **Microscopic** | | | | | **IHC** | **Prognosis** | |
| --- | --- | --- | --- | --- | --- | --- | --- | --- | --- | --- | --- |
|  |  |  |  | **Pathological pattern** | **Mitotic count**  **(n/10 HPFs)** | **Atypia** | **Necrosis** | **lymphatic metastasis** |  | **State** | **Time**  **(month)** |
| 1 | NA | NA | NA | NA | NA | NA | NA | No | NA | NA | NA |
| 2 | IB (2009 FIGO for uterine sarcomas) | total abdominal hysterectomy with bilateral salpingo-oophorectomy + chemotherapy | yellow-brown polypoid neoplasm | sarcoma | 15 | moderate | Yes | No | Pan-TRK,S100,SMA,H3K27me3(+);ER, PR, desmin, SOX10, CD34 (-) | metastatic  (vagina) | 7 |
| 3 | IB (2009 FIGO for uterine sarcomas) | total abdominal hysterectomy with bilateral salpingo-oophorectomy +chemotherapy | yellow-brown polypoid neoplasm | sarcoma | 12 | moderate | Yes | No | Pan-TRK,S100,SMA,H3K27me3(+);ER, PR, desmin, SOX10, CD34(-) | metastatic  (lung, pancreatic and brain) | 12 |
|  |  |  |  |  |  |  |  |  |  | pass away | 78 |
| 4 | IB (2009 FIGO for uterine sarcomas) | total abdominal hysterectomy with bilateral salpingo-oophorectomy | brown, threaded neoplasm | sarcoma | 30 | severe | No | No | Pan-TRK,S100,SMA,H3K27me3(+); ER, PR, desmin, SOX10, CD34(-) | survive | 2+ |
| 5 | IB (2009 FIGO for uterine sarcomas) | total abdominal hysterectomy with bilateral salpingo-oophorectomy | yellow-brown threaded nodules | sarcoma | 7 | moderate | No | No | Pan-TRK,S100,SMA,H3K27me3(+);ER, PR, desmin, SOX10, CD34(-) | survive | 11+ |
| 6 | IB1(NA) | radical abdominal hysterectomy, bilateral salpingectomy | NA | sarcoma | 2 | severe | No | No | CD34,S100,p16,Ki67,CD10 (+); SOX10(-) | survive | 4+ |
| 7 | NA | NA | NA | sarcoma | 3 | mild | Yes | No | TRK, S100, CD34 (+); ER, PR, desmin (-) | NA | NA |
| 8 | IA (2009 FIGO  for uterine sarcomas) | NA | NA | sarcoma | 3 | moderate | No | No | TRK, S100, CD34 (+); ER, PR, desmin (-) | survive | 2+ |
| 9 | IA (2009 FIGO  for uterine sarcomas) | NA | NA | sarcoma | 5 | moderate | Yes | No | TRK, S100, CD34 (+); ER, PR, desmin (-) | survive | 33+ |
| 10 | IA (2009 FIGO  for uterine sarcomas) | NA | NA | sarcoma | 50 | mild | No | No | TRK, S100, CD34 (+); ER, PR, desmin (-) | survive | 12+ |
| 11 | IIA (2009 FIGO  for uterine sarcomas) | NA | NA | sarcoma | 50 | mild | Yes | No | TRK, S100, CD34 (+); ER, PR, desmin (-) | metastatic  (omentum, small intestine, ovaries, liver) | 30 |
| 12 | IA (2009 FIGO  for uterine sarcomas) | NA | NA | sarcoma | 1 | mild | No | No | TRK, S100, CD34 (+); ER, PR, desmin (-) | survive | 108+ |
| 13 | IB (2009 FIGO  for uterine sarcomas) | NA | NA | sarcoma | 3 | moderate | Yes | No | TRK, S100, CD34 (+); ER, PR, desmin (-) | metastatic  (vagina) | 52 |
| 14 | NA | NA | NA | NA | NA | NA | NA | NA | pan-TRK (+) | NA | NA |
| 15 | NA | NA | NA | NA | NA | NA | NA | NA | pan-TRK (+) | NA | NA |
| 16 | NA | surgery (Details cannot be obtained) | yellow-pink, cystic changes and calcification | sarcoma | 0 | mild | No | NA | S100,CD34,Pan-TRK(+);SMN, perineurial, neuroendocrine, keratins, CD10(-) | survive | 36+ |
| 17 | I (NA) | polypectomy | pink-brown neoplasm | sarcoma | 0 | mild to moderate | No | No | Pan-TRK, S100, H3K27me3, Rb, p53(+); desmin, SMA, SOX10, caldesmon (-) | survive | 6+ |
| 18 | I (NA) | hysterectomy, bilateral salpingectomy, and pelvic lymph node dissection | pink-brown neoplasm, invasion half of the cervical wall | sarcoma | 5~18 | mild to moderate | No | No | Pan-TRK, S100, H3K27me3, Rb, p53 (+); desmin, SMA, SOX10, Caldesmon (-) | survive | 19+ |
| 19 | I (NA) | hysterectomy, bilateral salpingectomy, pelvic lymph node dissection + chemotherapy + pelvic radiotherapy+ larotrectinib | pink-brown neoplasm, invasion more than half of the cervical wall | sarcoma | 16~20 | mild to moderate | No | LVSI | Pan-TRK, S100, H3K27me3, Rb, p53 (+); desmin, SMA, SOX10, Caldesmon (-) | metastatic  (pleural) | 16 |
| 20 | NA | hysterectomy + adjuvant pelvic radiotherapy + brachytherapy | NA | sarcoma | 8 | NA | No | NA | Pan-TRK,CD10,vimentin,CyclinD1, CD34(+); Cytokeratins, desmin, SMA, caldesmon, ALK1, S100, SOX10 (-) | survive | 11+ |
| 21 | NA | hysterectomy and bilateral salpingo-oophorectomy | NA | sarcoma | 15 | mild | No | No | Pan-TRK,S100,CD34,SMA(+);keratins, ER, PR, SOX10, desmin, Cyclin D1 (-) | NA | NA |
| 22 | NA | hysterectomy, bilateral salpingo-oophorectomy and omentectomy | no mass in the cervix, only a circular, poorly defined white-brown fibrotic parenchyma | sarcoma | 0 | mild to moderate | No | NA | S100, CD34, SMA, ER, PR, H3K27me3 (+); pan-cytokeratin, epithelial membrane antigen, desmin, myogenin, SOX10, Melan-A, ALK1, neuro-Filament (-) | survive | 8+ |
| 23 | NA | tumor excision +larotrectinib +chemotherapy | NA | basaloid squamous cell carcinoma | NA | severe | Yes | NA | Collagen IV, P16(+); CK7, CK19, EMA, S-100, SMA, ER, PR, CD56 (-) | survive | 23+ |
| 24 | IIA2(2018 FIGO for cervical cancer) | hysterectomy and bilateral salpingo-oophorectomy +  pelvic lymph node dissection  +chemotherapy | pink and rich blood supply neoplasm in cervix | sarcoma | ＞10 | severe | No | No | S100,CD34,Pan-TRK,vimentin(+); SOX10,desmin,ALK,Caldesmon, MyoD1, Myogenin (-) | metastatic  (brain, lung and vagina) | 10 |
|  |  |  |  |  |  |  |  |  |  | pass away | 12 |

NA: not available in the original article. HPF: high power fields; IHC: immunohistochemistry; LVSI: lymphovascular space invasion.
